# Adipokines in Non-Alcoholic Fatty Liver Disease: Are We on the Road toward New Biomarkers and Therapeutic Targets?

**DOI:** 10.3390/biology11081237

**Published:** 2022-08-19

**Authors:** Vera Francisco, Maria Jesus Sanz, José T. Real, Patrice Marques, Maurizio Capuozzo, Djedjiga Ait Eldjoudi, Oreste Gualillo

**Affiliations:** 1 Institute of Health Research INCLIVA, Av. Menéndez Pelayo 4, 46010 Valencia, Spain; 2 Endocrinology and Nutrition Service, University Clinic Hospital of Valencia, Av. Blasco Ibañez 17, 46010 Valencia, Spain; 3 Department of Pharmacology, Faculty of Medicine and Odontology, University of Valencia, Av. Blasco Ibáñez 15, 46010 Valencia, Spain; 4 CIBERDEM-Spanish Biomedical Research Centre in Diabetes and Associated Metabolic Disorders, ISCIII, Av. Monforte de Lemos 3-5, 28029 Madrid, Spain; 5 Department of Medicine, Faculty of Medicine and Odontology, University of Valencia, Av. Blasco Ibáñez 15, 46010 Valencia, Spain; 6 National Health Service, Local Health Authority ASL 3 Napoli Sud, Department of Pharmacy, Ercolano, 80056 Naples, Italy; 7 The NEIRID Group (Neuroendocrine Interactions in Rheumatology and Inflammatory Diseases), SERGAS (Servizo Galego de Saude) and IDIS (Instituto de Investigación Sanitaria de Santiago), C027-Research Laboratory 9, Santiago University Clinical Hospital, 15706 Santiago de Compostela, Spain

**Keywords:** adipokines, NAFLD, liver, steatosis, inflammation, fibrosis, biomarkers, therapy, clinical trials, preclinical studies

## Abstract

**Simple Summary:**

Non-alcoholic fatty liver disease (NAFLD) is an unmet medical need due to its increasingly high incidence, severe clinical consequences, and the absence of feasible diagnostic tools and effective drugs. This review summarizes the preclinical and clinical data on adipokines, cytokine-like hormones secreted by adipose tissue, and NAFLD. The aim is to establish the potential of adipokines as diagnostic and prognostic biomarkers, as well as their potential as therapeutic targets for NAFLD. The limitations of current research are also discussed, and future perspectives are outlined.

**Abstract:**

Non-alcoholic fatty liver disease (NAFLD) has become the major cause of chronic hepatic illness and the leading indication for liver transplantation in the future decades. NAFLD is also commonly associated with other high-incident non-communicable diseases, such as cardiovascular complications, type 2 diabetes, and chronic kidney disease. Aggravating the socio-economic impact of this complex pathology, routinely feasible diagnostic methodologies and effective drugs for NAFLD management are unavailable. The pathophysiology of NAFLD, recently defined as metabolic associated fatty liver disease (MAFLD), is correlated with abnormal adipose tissue–liver axis communication because obesity-associated white adipose tissue (WAT) inflammation and metabolic dysfunction prompt hepatic insulin resistance (IR), lipid accumulation (steatosis), non-alcoholic steatohepatitis (NASH), and fibrosis. Accumulating evidence links adipokines, cytokine-like hormones secreted by adipose tissue that have immunometabolic activity, with NAFLD pathogenesis and progression; however, much uncertainty still exists. Here, the current knowledge on the roles of leptin, adiponectin, ghrelin, resistin, retinol-binding protein 4 (RBP4), visfatin, chemerin, and adipocyte fatty-acid-binding protein (AFABP) in NAFLD, taken from preclinical to clinical studies, is overviewed. The effect of therapeutic interventions on adipokines’ circulating levels are also covered. Finally, future directions to address the potential of adipokines as therapeutic targets and disease biomarkers for NAFLD are discussed.

## 1. Introduction

Non-alcoholic fatty liver disease (NAFLD) encompasses a spectrum of liver abnormalities characterized by increased hepatic fat content (>5%)—steatosis—in the absence of secondary causes, namely excessive alcohol consumption (>20 g/day for women and >30 g/day in men), medications, viral hepatitis, or certain hereditable conditions. Histologically, NAFLD can be categorized into non-alcoholic fatty liver (NAFL), when there is only evidence of hepatic steatosis, and non-alcoholic steatohepatitis (NASH), when, apart from steatosis, lobular inflammation and hepatocyte ballooning with or without perisinusoidal fibrosis can be observed. NASH, especially in the fibrotic state, often presents worse prognoses and frequently progresses to more severe conditions, such as cirrhosis and hepatocellular carcinoma [1].

NAFLD has become the major cause of chronic hepatic illness in children and adults, as well as the leading indication for liver transplantation in the future decades, replacing chronic hepatitis C. This high-incident pathology is also associated with extra-hepatic complications, such as chronic kidney disease and cardiovascular disease, which is the leading cause of death among NAFLD patients [2]. Mainly asymptomatic, NAFLD is considered a major societal, clinical, and research challenge due to its increasingly high prevalence, difficulties in its diagnosis, the lack of approved therapies, and its complex pathophysiology.

The development and progression of NAFLD is induced by multiple factors in a “multiple parallel-hit” model, where numerous genetic and environmental determinants (“hits”) interplay on an individual basis. These factors encompass, but are not limited to, genetic alterations, inflammation, gut dysbiosis, and metabolic abnormalities. Indeed, almost 90% of NAFLD patients present at least one of the metabolic syndrome features (abdominal obesity, hypertriglyceridemia, low HDL-cholesterol, hypertension, and high fasting glucose), and about 33% fulfill the criteria for diagnosing metabolic syndrome [3]. Therefore, metabolic-associated fatty liver disease (MAFLD) was recently proposed as a more appropriate overarching term that recognizes the metabolic risk profile of patients as the master criterion for diagnosis and considers the disease activity grade to be a continuum, which better describes disease pathophysiology [4]. Therefore, it is not surprising that the worldwide prevalence of NAFLD is rising in parallel with obesity [2], the main driver of metabolic abnormalities. Stepping up the strict correlation between obesity and NAFLD, there is a dose-response between weight loss and histological disease improvement; a 7% weight loss reverts NASH in 65–90% of patients, and a ≥10% weight loss causes fibrosis relapse in 45% of patients [3].

In the last 10 years, researchers have gained substantial insights into the role of adipose tissue in NAFLD. Initially seen as a simple energy storage tissue, adipose tissue is now recognized as an active endocrine organ, the dysfunction of which impacts either the initial stages of liver steatosis or the progression to NASH and fibrosis [5]. Adipokines, cytokine-like hormones secreted by adipose tissue, have emerged as cornerstone players in the regulation of energy metabolism, inflammation, and fibrosis [6,7], making them potential therapeutic targets for NAFLD. Furthermore, a vast number of clinical trials have established the serum profiles of classical (leptin and adiponectin) and emergent adipokines, such as ghrelin, resistin, retinol binding protein 4 (RBP4), visfatin, chemerin, and adipocyte fatty acid-binding protein (AFABP), in NAFLD patients [8].

In this review, we briefly summarize the main aspects of the adipose tissue–liver communication driving NAFLD pathophysiology. Following this, we overview the current knowledge on the role of adipokines in the development and progression of NAFLD and present data from clinical studies on adipokine profile in NAFLD patients. Moreover, we cover the effect of therapeutic interventions on circulating adipokines in the context of the management of NAFLD-associated metabolic complications. Finally, we discuss future directions to address the potential of adipokines as therapeutic targets and disease biomarkers for NAFLD.

## 2. NAFLD, an Unmet Medical Need

Over the last century, dramatic changes in lifestyle behaviors have evoked a growing incidence of noncommunicable diseases. The Global Burden of Disease Study 2017 estimated the prevalence of NAFLD for the first time, reflecting that the emerged epidemic in chronic liver disease is related to the burden of this pathology [9]. Parallel to the worldwide augment of obesity, the global prevalence of NAFLD is currently estimated to be 24%, with South America and the Middle East having the highest rates, followed by Asia, the USA, and Europe [2]. The clinical consequences of NAFLD, particularly its histological phenotype NASH, which likely progresses to cirrhosis and hepatocellular carcinoma, are the second-most common indication of liver transplantation in the USA [2]. NAFLD also potentially contributes to the burden of extra-hepatic chronic complications, namely cardiovascular disease, type 2 diabetes (T2DM), and chronic kidney disease [2]. Aggravating the health and economic consequences of NAFLD, weight-management difficulties, together with unavailability of routinely feasible diagnostic procedures and effective pharmacotherapeutic approaches, challenged the increasing need of the management of this complex and frequently asymptomatic pathology.

The “gold standard” method for diagnosing NASH (and differentiation from NAFL) is liver biopsy, even though it has limitations related to invasiveness, patient discomfort, risk of adverse events, sampling variability, pathologist experience, and unsuitability for large-scale screening, as well as its debatable cost-effectiveness needs, since approved NASH-specific therapies are not presently available [3]. A non-invasive multistep approach for NAFLD diagnosis and follow up, which considers patients clinical history (patients with obesity and/or metabolic syndrome or T2DM are at high risk of NAFL/NASH), imaging techniques (such as ultrasonography and transient elastography), biomarkers, and scoring systems to estimate steatosis or fibrosis (NAFLD Activity Score or NAS, Fibrosis-4 index or FIB-4, NAFLD Fibrosis Score or NFS, and Aspartate aminotransferase to Platelet Ratio Index or APRI), is a useful tool to guide disease management. However, these predictive approaches have decreased sensitivity and specificity to the earlier and more moderate stages of fibrosis and have broad diagnostic grey zones [10]. Therefore, liver biopsy remains the only reliable diagnostic tool for NAFL/NASH identification and assessing fibrosis severity.

Despite the increasingly high incidence of NAFLD worldwide, its associated morbidity and mortality, and the intensive research on the field, there is no specific drug approved for its treatment, and lifestyle change remains the cornerstone of NAFLD management, as indicated by clinical guidelines [11]. If exercise and diet fail to achieve the targets in NAFLD patients, bariatric surgery or pharmacotherapy to manage the underlying metabolic complications, namely obesity, T2DM, dyslipidemia and cardiovascular disease, is recommended [12]. The current and emerging pharmaceutical therapeutic strategies for NASH aim to improve metabolic function, reduce steatosis, decrease inflammation, and halt or reverse the progression of fibrosis. Among the extensive list of potential pharmacotherapies are those targeting oxidative stress (vitamin E), insulin resistance (IR) or high glucose blood levels (thiazolidinediones, metformin and sodium-glucose cotransporter-2 inhibitors), and dyslipidemia/lipotoxicity (statins). Nevertheless, to date, no molecule studied for the treatment of NAFLD has demonstrated convincing effects on halting or reverting liver disease [13].

## 3. Adipose Tissue-Liver Axis in NAFLD Pathophysiology

Adipose tissue (AT) and the liver play a critical role in the regulation of whole-body energy homeostasis. Triglycerides (TG) and excess carbohydrates from the diet are converted into fatty acids (FA) in the liver. In particular, dietary TG are emulsified by bile acids and hydrolyzed by pancreatic lipase, yielding FA, while excess carbohydrates are converted into FA by *de novo* lipogenesis (DNL). Hepatic FA are then esterified and stored in cytoplasmatic lipid droplets or secreted into bloodstream. Circulating FA are uptaken and used as energy sources by brown adipose tissue and muscle or re-esterified to TG and stored in white adipose tissue (WAT). In WAT, insulin-stimulated FA uptake and TG synthesis inhibits the intracellular lipolysis of cytosolic TG, thereby promoting adipocyte TG storage [14].

In overweight and obese subjects, the capacity for WAT expansion reaches its limit; therefore, energy cannot be stored in WAT, and ectopic lipid accumulation occurs in other tissues, including the liver. Resistance to insulin also promotes lipolysis in adipocytes with a further increase of circulating free FA. Consequently, intrahepatic fat accumulation (steatosis) develops, which can be further amplified by FA synthesis from excess carbohydrates through DNL. At this stage, TGs-derived toxic metabolites can cause hepatic lipotoxicity, this results in cellular dysfunction, lipoapoptosis, IR, and the activation of hepatic innate immune cells, including dendritic cells, Kupffer cells, and hepatic stellate cells (HSCs); all of these are essential features of NASH [15].

Beyond its energy-storage functions, WAT is a recognized active endocrine and an immunological organ constituted by mature and developing adipocytes, endothelial cells, fibroblasts, and immune cells that maintain tissue homeostasis, such as AT macrophages, eosinophils, mast cells, neutrophils, and T and B cells [16]. In the WAT of lean subjects, eosinophils and T regulatory cells (Treg) secrete anti-inflammatory cytokines, such as interleukin (IL)-4 and IL-10, that polarize AT macrophages towards the M2 phenotype (anti-inflammatory phenotype), thus preserving a tolerogenic environment [17]. Adipocyte expansion derived from TG accumulation is accompanied by adipocyte hypoxia, cell stress, and apoptosis, able to promote chemoattractant molecules’ expression and immune-cell infiltration. The immune profile of WAT is then modified; T cells become activated, the presence of T reg is reduced, and the macrophage’s phenotype switches from M2 to M1 (pro-inflammatory phenotype), which accumulates around necrotic adipocytes forming ‘crown-like structures’ and producing pro-inflammatory cytokines, such as tumor necrosis factor (TNF)-α and IL-6 [16]. Of relevance, this pro-inflammatory profile is correlated with local and systemic IR [18].

In the WAT of obese subjects, a deregulated production of cytokine-like hormones, known as adipokines, is observed. This family of low-molecular-weight, bioactive peptides has a proven pleiotropic function as hormones and cytokines with both pro- and anti-inflammatory activities. Namely, adipokines show a fundamental role in energy metabolism by communicating the nutrient status of the body through the induction of anorexigenic signals and the suppression of orexigenic factors at the hypothalamus [19]. Adipokines have also been revealed to be critical regulators of hepatic lipogenesis and insulin sensitivity; for example, adiponectin has been shown to augment insulin sensitivity, maintain healthy AT expansion, and rescue the body from ectopic lipid accumulation [20]. Moreover, adipokines were highlighted as cornerstone modulators of both the innate and adaptive immune systems. For instance, leptin, the first adipokine to be discovered, modulates both the innate immune response, by increasing natural killer cells cytotoxicity and modulating granulocytes, macrophages, or dendritic cell activation, and the adaptive immunity, by increasing naïve T and B cell proliferation, promoting a switch towards a pro-inflammatory Th1 phenotype, and reducing Treg proliferation [6]. The deregulated production of adipokines thus contributed to obesity-associated systemic chronic low-grade inflammation, and the liver is progressively infiltrated by immune cells, such as monocytes, neutrophils, and T cells. When inflammation perpetuates, fibrogenesis starts with the progression to NASH with fibrosis, the most severe grade of the disease [21].

**Figure 1:**
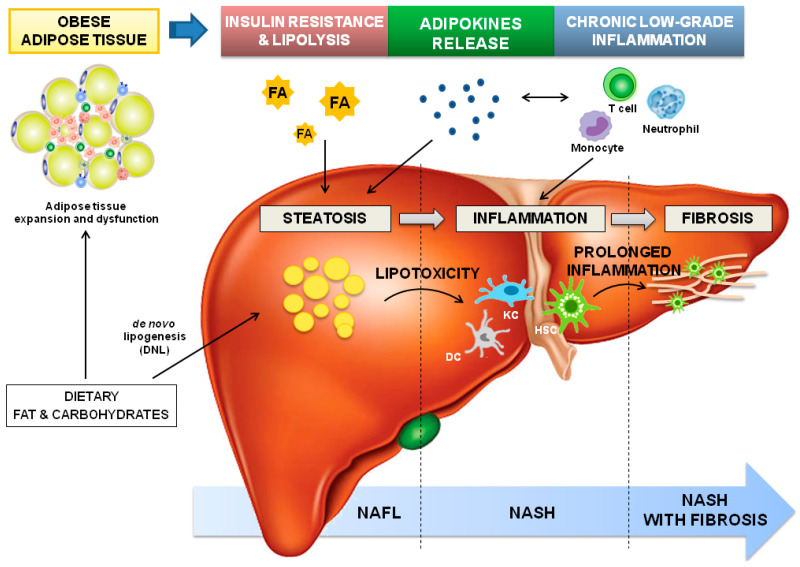
**Dysfunctional adipose tissue-driven pathophysiology of NAFLD.** The expansion of white adipose tissue in obesity induces adipocyte dysfunction and insulin resistance leading to lipolysis. As a consequence, circulating fatty acid (FA) and adipokine imbalance towards pro-steatogenic ones contribute to intrahepatic fat accumulation (steatosis), which is amplified by the high dietary fat and carbohydrate levels (commonly observed in obesity), the latter augmenting de novo lipogenesis (DNL). The ectopic accumulation of triglycerides (TGs)-derived toxic metabolites induces lipotoxicity and the consequent cellular dysfunction (endoplasmic reticulum and oxidative stress, and mitochondrial defects), lipoapoptosis and activation of inflammatory pathways. The expansion of adipocytes also elevates the expression of chemoattractants, and immune cells infiltration, and promotes deregulation of adipokines secretion; altogether, contributing to systemic chronic low-grade inflammation. Additionally, in the liver, dendritic cells (DC), Kupffer cells (KC), and hepatic stellate cells (HSCs) are activated, and hepatic infiltration by circulating immune cells, including neutrophils, monocytes, and T-lymphocytes, occurs. When inflammation perpetuates, fibrogenesis starts, with HSCs as key players. Abbreviations: DC-Dendritic cells; DNL-de novo lipogenesis; FA-Fatty acids; HSC-Hepatic stellate cells; KC-Kupffer cells; NAFL-Non-alcoholic fatty liver; NASH-Non-alcoholic steatohepatitis; TGs- triglycerides.

In summary, the adipose tissue–liver axis is essential for the regulation of glucose and lipid metabolism, as well as immunological homeostasis, and a dysfunctional secretion of adipokines from WAT could (i) determine the fluxes of lipid to the liver and, thus, hepatic steatosis; and (ii) promote hepatic inflammation (characteristic of NASH) that, if not managed in a timely fashion, can progress to hepatic fibrosis (Figure 1). In this context, adipokines (overviewed in Table 1) have been identified as potential therapeutic targets and investigated as diagnostic and prognostic biomarkers of NAFLD. The following sections review the current knowledge on adipokines in NAFLD, focusing on their activity on NAFLD pathophysiological mechanisms, namely IR, hepatic lipid accumulation, inflammation, and fibrosis. Moreover, clinical studies establishing the serum profiles of adipokines in NAFLD and therapeutic approaches used in the management of NAFLD and affecting adipokines levels in humans were described. Since very recent reviews summarized the action of leptin [22,23] and adiponectin [24,25] in NAFLD in detail, here we will focus on the preclinical and clinical data of less-studied adipokines (ghrelin, resistin, RBP4, visfatin, chemerin, and AFABP) in NAFLD, briefly overviewing the activity of leptin and adiponectin.

## 4. Adipokines as Potential Therapeutic Targets for NAFLD

### 4.1. Leptin and Adiponectin

Leptin, the forerunner and best-characterized member of the adipokine family, plays a pivotal role in appetite and body-weight homeostasis by augmenting anorexigenic neuropeptides and suppressing orexigenic factors in the central nervous system [6]. Likewise, leptin has been described as modulating several physiological processes, such as lipid and glucose metabolism, as well as both innate and adaptive immunity [6]. Most of the current knowledge about leptin’s action arose from leptin-deficient *ob/ob* mice and leptin-receptor-deficient *db/db* mice. These murine models exhibited marked hepatic alterations, such as IR, accumulation of TG and lipids, and steatosis, which were partially reverted following leptin administration to *ob/ob* mice [22,23]. Although leptin acts as an anti-steatotic hormone preventing the accumulation of and promoting the mobilization of hepatic lipids, leptin resistance may cause the leptin inability to alleviate hepatic steatosis [22,23]. Furthermore, high leptin plasma levels derived from obese adipose tissue are associated with hepatic inflammatory and fibrogenic mechanisms and therefore, with NAFLD development [22,23].

On the contrary, adiponectin, an adipocyte-secreted hormone inversely correlated with obesity and with determining roles in insulin sensitivity, glucose levels, and lipid metabolism [25], has been reported to protect the liver from steatosis, inflammation, and fibrosis [24]. Adiponectin augments insulin’s capacity to suppress glucose production, prevents hepatic DNL, suppresses FA synthesis in hepatocytes, and enhances FA β-oxidation [24], overall protecting the liver from steatosis. Adiponectin was also described as decreasing the production of inflammatory cytokines, such as IL-6 and TNF-α, through the modulation of toll-like receptor 4 (TLR4) signaling. By boosting the beneficial effects of adiponectin in NAFLD development, this adipokine was described to possess anti-fibrotic effects by preventing leptin profibrogenic signaling [24]. Therefore, strategies aiming to rescue the leptin–adiponectin balance, i.e., reverting the obesity-associated increased leptin levels and reduced adipokines levels, are of relevance for NAFLD treatment [24]; this will be further explored in Section 5.

### 4.2. Ghrelin

Ghrelin stands out as one of the few peripheral peptide hormones with an orexigenic effect. Initially identified as a stomach-derived hormone, ghrelin is an endogenous ligand for the growth hormone secretagogue receptor 1a (GHSR1a). It stimulates food intake and adiposity and acts as a regulator of glucose metabolism, reward behavior, gut motility, or even hepatic lipid metabolism and the immune system [26,27]. This hormone circulates in blood in two forms, an acylated (AG) form and an unacylated form (UAG, also named DAG from desacyl ghrelin). This post-transcriptional modification is catalyzed by ghrelin O-acyltransferase (GOAT), and the producing AG is the active form that triggers the signaling of the cognate receptor GHSR1a. Initially thought to be an inactive form, it has been suggested that UAG to antagonizes AG activity on glucose metabolism and lipolysis and reduces food consumption and body weight [27,34]. Given its regulatory activity on metabolism and immune system, there is a growing interest on the role of ghrelin-GOAT system in the development and progression of NAFLD.

In murine models, the administration of ghrelin during or after diet-induced NAFLD development counteracts dysregulated hepatic lipid metabolism, oxidative stress, apoptosis, and inflammation [35,36]. Furthermore, the action mechanisms involved in the beneficial effects of ghrelin on NAFLD were investigated. The amelioration of liver injury by ghrelin was accompanied by the reestablishment of phosphoinositide 3-kinase (PI3K)/Akt and liver kinase B1 (LKB1)/AMP-activated protein kinase (AMPK) pathways [35]. Ghrelin also attenuates lipotoxicity by upregulating autophagy via AMPK/mTOR restoration and inhibiting nuclear factor kappa B (NF-κB) [36]. Recently, ghrelin was demonstrated to block the progression of NASH induced by lipopolysaccharide (LPS) in mice fed with a high-fat diet through the reduction of Kupffer cells’ M1 polarization, which is mediated by GHSR1a [37]. In addition to these results demonstrating the beneficial effects of ghrelin, the genetic deletion of ghrelin in mice also significantly reduced age-associated hepatic steatosis, partly by downregulating diacylglycerol O-acyltransferase-1, one of the key enzymes of TG synthesis [38].

Concerning the effects of the ghrelin’s isoforms, it was verified that plasma UAG levels were reduced after a sleeve gastrectomy and a Roux-en-Y gastric bypass in Wistar rats, whereas the AG:UAG ratio was augmented. Concomitantly, both surgeries diminished obesity-associated hepatic steatosis, inflammation, mitochondrial dysfunction, and endoplasmic reticulum stress. Moreover, AG has a similar effect in palmitate-treated hepatocytes, which suggests that the increased AG:UAG ratio after bariatric surgery might ameliorate obesity-associated NAFLD [39]. Possible underlying mechanisms are the reduction of lipogenesis and stimulation of mitochondrial FA β-oxidation, as well as hepatic autophagy, by relatively increased AG levels [40]. Nevertheless, in lean rats, the administration of exogenous AG induced hepatic IR and lipid accumulation, while the co-administration of UAG prevented the AG-induced effects [41]. Thus, further evaluation of the ghrelin-GOAT system and the effects of AG and UAG isoforms on NAFLD development is needed.

### 4.3. Resistin

Resistin (named for its ability to induce “resistance to insulin”) is the founding member of resistin-like molecules (RELMs), a family of small, secreted cysteine-rich peptides with hormone-like and pro-inflammatory activities. It is mainly secreted by adipose tissue and inflammatory cells, and its action is thought to be mediated by the TLR4 receptor, although the receptors tyrosine kinase-like orphan receptor (ROR)-1, insulin-like growth factor type 1 receptor (IGF-1R), and adenylyl cyclase-associated protein 1 (CAP1) have also emerged as potential candidate receptors [28]. Resistin has been shown to have pleiotropic effects, including the regulation of blood–glucose levels and lipid metabolism, as well as the induction of pro-inflammatory cytokines secretion or monocyte differentiation into macrophages [28]. In fact, resistin administration to C57BL/6J mice increased blood glucose, i.e., impaired glucose tolerance, due to the decreased insulin sensitivity, which was rescued after the administration of an anti-resistin antibody [42]. Similarly, the high-fat diet (HFD)-fed mice presented increased plasma resistin levels and severe IR, which is completely reversed after treatment with a resistin antisense oligonucleotide [43]. More recently, the mechanisms of action of resistin in a HFD-induced NAFLD model were disclosed [44]. Acute elevated resistin altered mitochondrial morphology and content, increased lipid accumulation, and up-regulated pro-inflammatory mediators in HFD-fed mice and palmitate-treated HepG2 cells. Furthermore, steatosis aggravation induced by resistin in mice is mediated by the AMPK/peroxisome proliferator-activated receptor gamma coactivator 1-alpha (PGC-1α) pathway [44]. It was also reported that resistin treatment augments the suppressor of cytokine signaling 3 (SOCS3) expression, a suppressor of insulin signaling, in adipocytes [45].

Resistin-deficient mice demonstrated reduced hepatic glucose production and, consequently, their blood–glucose levels after fasting were low [46]. In addition, *ob/ob* mice and diet-induced obese mice, both lacking resistin, had reduced hepatic steatosis, since the expression of genes involved in hepatic lipogenesis and the secretion of very-low-density lipoprotein (VLDL) were decreased [47].

At the cellular level, resistin hampered glycogen synthase kinase 3β (GSK3β) phosphorylation in primary rat hepatocytes under hyperinsulinemic and hyperglycemic conditions, with further reduction of glycogen synthesis and hepatic insulin action [48]. Resistin also exerts pro-inflammatory activity by inducing the expression of cell adhesion molecules and pro-inflammatory cytokines in macrophages and mononuclear cells, contributing to the recruitment of leukocytes to inflammation sites [28]. Moreover, the secretion of resistin by infiltrated monocytes/macrophages is enhanced by pro-inflammatory mediators [28]. Of note, hepatic myeloid cells and T-lymphocytes from NAFLD patients showed a decreased response to resistin, which is associated with a failure to maintain redox homeostasis, which would be a risk factor for NAFLD severity [49]. It was also verified that resistin increased hepatic inflammation through mitogen-activated protein kinase (MAPK) signaling and the activation of a coagulation cascade in animal models [50]. Finally, resistin demonstrated profibrogenic effects by activating HSCs, which release IL8/CXCL8 and monocyte chemoattractant protein (MCP)-1/CCL2 via NF-κB, and increasing the transforming growth-factor beta (TGFβ) and collagen type I production in Kupffer cells [51,52].

### 4.4. Retinol Binding Protein 4 (RBP4)

Distinct mouse models have been used to elucidate the RBP4 activity in metabolic diseases. In general, elevated circulating and adipose tissue RBP4 levels have been correlated with IR, dyslipidemia, and T2DM [29]. The possible RBP4-dependent mechanisms contributing to IR include impaired insulin signaling, the down-regulation of GLUT-4 translocation, and the induction of inflammatory and lipolytic pathways in adipose tissue, as well as the induction of phosphoenolpyruvate carboxykinase in the liver, thereby increasing glucose production [29].

In genetic and dietary mouse models of NAFLD, the results of hepatic expression of RBP4 are controversial. Liu et al. observed an abnormal hepatic RBP4 expression in apoE−/− mice fed with a high-fat and high-cholesterol (HFC) diet [53]. However, Saeed et al. described reduced hepatic RBP4 levels in C57BL/6J mice fed with a HFC diet and in *ob/ob* mice, while serum RBP4 levels were increased in both models [54]. Apart from that, transgenic mice overexpressing human RBP4 had increased hepatic lipid accumulation, cellular ballooning, and inflammation, which were exacerbated when mice were challenged with a high-fat diet, likely through RBP4-induced mitochondrial dysfunction [53,55]. Of note, recently, novel RBP4 antagonists were reported to reduce hepatic steatosis in transgenic mice with adipocyte-specific overexpression of RBP4 [56].

### 4.5. Visfatin

Nicotinamide phosphoribosiltransferase (NAMPT), also called pre-B cell colony-enhancing factor (PBEF) or visfatin, functions as an intracellular enzyme (iNAMPT) mediating the synthesis of nicotinamide adenine dinucleotide (NAD+) and as a cytokine-like soluble factor secreted into extracellular space (eNAMPT) [31]. Intracellular NAMPT regulates mitochondrial biogenesis, cellular metabolism, and survival, as well as the adaptive response to cell stress; it was described as modulating pancreatic β-cell function, likely regulating glucose homeostasis and IR [30]. On the other hand, extracellular NAMPT acts mainly as an inductor of pro-inflammatory cytokine production [31]. The NAMPT extracellular form has been associated with metabolic and inflammatory disorders, but its pathophysiological mechanisms are still ill-defined [31].

Administration of NAMPT to a methionine-choline-deficient (MCD)-diet-fed mouse model of NAFLD aggravated hepatic steatosis, increased inflammatory cell infiltration and inflammatory cytokines levels and exacerbated the expression of fibrotic markers in the liver, together with the induction of endoplasmic reticulum and oxidative stress [57]. In hepatocytes, NAMPT also induced the expression of inflammatory cytokines and diminished insulin signaling through a signal transducer and activator of transcription 3 (STAT3) and NF-κB activation [58]. These results support the adverse effects of NAMPT in hepatic steatosis, inflammation, and fibrosis. However, the pharmacologic inhibition or genetic ablation of NAMPT also showed deleterious effects. The intracellular NAMPT inhibitor FK866 promoted liver steatosis in HFD-fed mice and hepatic lipid accumulation in vitro via the sirtuin 1 (SIRT1)/sterol regulatory element-binding protein 1 (SREBP1)/fatty acid synthase (FASN) pathway [59]. Similarly, the knockdown of NAMPT in hepatocyte cells led to TG accumulation through the regulation of DNL via SIRT1/AMPK pathway [60]. Accordingly, the overexpression of NAMPT in hepatocyte cell lines mitigated lipid accumulation [59]. However, hepatocyte-specific NAMPT knockout mice on low-methionine and choline-free high-fat diet showed less TG accumulation than wild-type controls but had augmented histological scores for hepatic inflammation, necrosis, and fibrosis [61]. Of note, NAMPT KO mice on a control diet also showed liver injury, since they had decreased mitochondrial proteins and respiratory capacity and increased fibrosis due to low NAD+ levels [61]. Taken together, the present data suggest that the opposing activity of NAMPT in NAFLD pathophysiology could be derived from the different roles of extracellular and intracellular NAMPT, but further studies are needed.

### 4.6. Chemerin

Chemerin, also named tazarotene-induced gene 2 (TIG2) and retinoic acid responder 2 (RARRES2), is secreted as inactive precursor, which is activated by proteases of the coagulation cascade, neutrophil-derived proteases (elastase and cathepsin G), bacterial proteases, and mast cell products (tryptase) [32]. This chemotactic adipokine binds to the G protein-coupled receptor chemokine-like receptor 1 (CMKLR1), which is expressed in dendritic cells, macrophages, and natural killer (NK) cells and may serve as bridge between innate and adaptive immunity [62]. Although the C-C chemokine receptor-like 2 (CCRL2) and the G protein-coupled receptor 1 (GPR1)/CMKLR2 were also described as chemerin receptors, their physiological activity is still uncertain. Chemerin and its receptor CMKLR1 are both expressed in adipose tissue [63], and they have been reported to be augmented in obesity and IR states (T2DM), decreasing after weight loss [64]. This adipokine also seems to regulate adipocyte differentiation, glucose and lipid homeostasis, and insulin sensitivity [32].

In addition to its ability to regulate glucose metabolism, IR, and inflammation, the role of chemerin in NAFLD is still unclear. The administration of recombinant chemerin ameliorate HFD-induced NASH in mice, as well as IR, leptin resistance, and liver lesions, by alleviating oxidative stress and promoting autophagy, at least in part, due to chemerin/CMKLR1-dependent activation of janus kinase 2 (JAK2)-STAT3 pathway [65]. On the contrary, the administration of a chemerin-derived C15 peptide did not affect hepatic TG accumulation, inflammation, or fibrotic gene expression in atherogenic diet-induced murine NASH [66]. Moreover, PI3K inhibition mitigated liver steatosis and KC-mediated inflammation due to the down-regulation of the chemerin receptor CMKLR1 in the liver [66]. Nevertheless, whole-body *Cmklr1*-gene abrogation in mice did not affect either the hepatic lipid accumulation, inflammation, fibrotic gene expression, NAS, or the IR [67]. Inconsistencies in the current data could be related to the differential modulation of hepatic chemerin in distinct murine models of NAFLD [68].

### 4.7. Adipocyte Fatty Acid-Binding Protein (AFABP)

There is strong evidence correlating elevated AFABP with IR and adipose tissue lipolysis in obesity and metabolic syndrome [33]. Interestingly, a recent review pointed out that AFABP as a metabolic/functional marker regulating macrophage functions likely having a determining role in pathophysiology [69]. Concerning to NAFLD, AFABP expression was elevated in Kupffer cells in both LPS-induced acute liver injury and diet-induced NAFLD [70]. In these NAFLD mice models, the pharmacological inhibition of AFABP ameliorated hepatic steatosis, macrophage infiltration, and hepatocellular ballooning [70]. Genetic ablation and the pharmacological inhibition of AFABP also attenuated bile-duct-ligation- and carbon-tetrachloride-induced liver fibrosis in mice through the reduction of collagen accumulation, liver sinusoidal endothelial cells (LSEC) capillarization, and HSC activation [71]. Mechanistically, elevated AFABP promotes LSEC capillarization, an early event of NAFLD pathogenesis, and LSEC-derived AFABP activate HSCs that augments TGFβ production and further extracellular matrix accumulation and fibrosis [71]. Furthermore, AFABP could promote hepatic inflammation through Kupffer cell activation [70]. Altogether, these findings suggest pharmacological inhibition of AFABP as a promising therapeutic strategy for NAFLD.

## 5. Adipokines in NAFLD: Evidence from Clinical Studies

### 5.1. Ghrelin

Clinical studies have demonstrated that obese patients with IR or metabolic syndrome had lower UAG and total ghrelin levels, while the AG:UAG ratio was elevated [72,73]. Moreover, the AG:UAG ratio was positively correlated with IR in both obese children and adults [72,74]. After the proof about the influence of ghrelin on insulin sensitivity, some research focused on ghrelin’s role in NAFLD, but the evidence is scarce, and not all studies take into consideration the concentrations of total ghrelin and its isoforms, AG and UAG.

Ghrelin levels were associated negatively with body mass index (BMI) in obese NAFLD patients diagnosed by ultrasonography [75] or biopsy [76]. Compared to matched healthy controls, patients with NAFLD showed reduced ghrelin levels, which correlated with IR [77]. However, in a study with NAFLD biopsy-proven patients that underwent bariatric surgery, UAG levels were increased in NASH patients compared to non-NASH subjects, while similar levels of AG were observed. Additionally, higher levels of AG, but not UAG, were observed in higher stages of fibrosis [78]. Further evidencing the role of ghrelin in NAFLD, recent case-control retrospective studies of biopsy-proven NAFLD patients suggested that the Leu72Met (rs696217 G > T) polymorphism and the “GG” genotype of rs26802 variant in the ghrelin gene have a protective effect against NAFLD [79,80].

### 5.2. Resistin

As described above, the positive correlation between resistin and IR, steatosis, and inflammation is well stablished in murine and cellular models, but data in human NAFLD are conflicting. A systematic review of Bekaert et al. found 12 studies reporting resistin levels in biopsy-proven NAFLD patients, of which 6 reported statistically significant results [81]. Circulating resistin levels were positively correlated with hepatic steatosis, portal inflammation, and NAFLD ACTIVITY SCORES in non-diabetic NAFLD patients [82,83,84,85]. Of relevance, Aller et al. found that resistin association with the steatosis grade was lost when the homeostatic model assessment of insulin resistance (HOMA-IR) parameter was included in the multivariate logistic analysis, indicating that resistin is a surrogate marker of IR [83]. Supporting the relevance of resistin in NAFLD, a predicting diagnostic biomarker panel for histological NASH in obese subjects included the serum levels of resistin together with adiponectin and cytokeratin 18 (marker of cell death) [86]. However, resistin was not included in the predicting algorithm for NASH or NASH-related fibrosis in a more recent study by the same group [87]. In contrast, a study described a negative correlation between circulating resistin levels and the steatosis grade in severely obese NAFLD patients [88]. The remaining studies included in the cited meta-analysis did not demonstrate an association of resistin with liver histological parameters in obese and non-obese NAFLD patients [89,90,91,92]. It is worth mentioning that, in this meta-analysis, 4 out of the 12 studies did not adjust their results for confounding factors, and the potential association between resistin and IR was conflicting among the studies [81]. More recently, the determination of serum resistin levels in severe obese NAFLD patients found no correlation with steatohepatitis or fibrosis severity [93]. Similarly, resistin circulating levels did not associate with steatosis grade, NASH diagnosis, hepatic ballooning, or lobular inflammation grade, but they did correlate with fibrosis stage in obese NAFLD patients [94].

Although there are ambiguous data on circulating resistin levels in NAFLD patients, the existing results on its hepatic expression are more consistent. Augmented hepatic resistin mRNA levels were reported in patients with NASH compared to steatosis or control subjects and in steatosis patients compared to control individuals [95]. Moreover, a positive association between hepatic resistin mRNA levels and hepatic steatosis, inflammation, or fibrosis was reported in several studies [95,96]. Additionally, a positive correlation between hepatic resistin protein expression and NAS, aspartate aminotransferase (AST), alanine aminotransferase (ALT), BMI, glucose, insulin, HOMA-IR, gamma-glutamyl transferase (GGT), lactate dehydrogenase (LDH), TG, and glycated haemoglobin was verified in obese NAFLD patients [97]. The resistin mRNA expression in subcutaneous adipose tissue was also verified to be increased in non-obese NAFLD patients, but no correlation with liver histological parameters was observed [84]. Interestingly, the immuno–histological assessment of hepatic samples of NAFLD patients revealed a resistin distribution predominantly in perisinusoidal cells (Kupffer cells and HSCs) [95], histiocytes of inflammatory infiltrate, and histiocytes surrounding the hepatocytes with steatosis [97]. Finally, a significant association was observed between resistin rs1862513 polymorphism and NAFLD [98], which supports the significance of resistin in the development of NAFLD.

### 5.3. Retinol Binding Protein 4 (RBP4)

Several clinical studies showed that elevated circulating RBP4 levels were associated with insulin-resistance states, namely obesity and T2DM [29]. In addition, decreased levels of RBP4 were correlated with recovering insulin sensitivity after weight loss or lifestyle intervention in obese adult or children populations [99,100]. Given the close association of NAFLD pathogenesis and IR, NAFLD is assumed to be correlated with increased levels of serum RBP4; however, inconsistent findings were observed. In studies without histological confirmation, serum RBP4 levels seemed to be positively correlated with liver fat [101] and were found to be higher in NAFLD patients than controls, in adult and pediatric subjects [102,103]. Nevertheless, a systematic review reported that only three out of seven studies verified a positive correlation between serum RBP4 levels and liver histology among patients with biopsy-proven NAFLD [81]. Similarly, a meta-analysis research did not find any significant differences between NAFLD, NASH, or SS patients compared to controls, neither between NASH nor SS patients [104]. The authors highlighted the heterogeneity across patient populations or the lack of adjustment for confounding factors in the analyzed research, which challenges comparisons between studies and limits the conclusions that can be drawn about the associations between adipokines levels and NAFLD. More recently, a 3-year follow-up study in a Chinese cohort of NAFLD patients diagnosed by abdominal ultrasonography verified that baseline serum RBP4 concentrations are positively associated with NAFLD development and inversely correlated with NAFLD regression [105]. Moreover, higher serum RBP4 levels were associated with an increased risk for prediabetes and metabolic syndrome in obese patients with NAFLD [106].

### 5.4. Visfatin

Several studies have evaluated the levels of visfatin in histologically confirmed NAFLD patients as well as the possible correlations with hepatic steatosis, inflammation, and fibrosis; but, current data are limited and inconclusive, as verified by two systematic reviews [81,107]. Most data reported similar serum visfatin levels in NAFLD [108], simple steatosis (SS) [109], or NASH patients [109,110] compared to control subjects, as well as in NASH compared to SS patients [109,111]. Likewise, similar hepatic visfatin expression was found in NASH and SS patients [112]. However, some studies also verified the augmented levels of serum visfatin in NAFLD compared to controls [113] or the decreased visfatin levels in NAFLD [114], SS, or NASH patients versus controls [86,111]. Of note, increased serum visfatin levels were associated with a reduced hepatic DNL index in women with ultrasound-diagnosed NAFLD, while in men it was correlated with augmented hepatic fat but not with DNL index, which suggests a sex-dependent interpretation for the serum visfatin levels in NAFLD prognosis [115]. 

Concerning histological parameters, most data did not report any correlation between serum visfatin and hepatic steatosis, inflammation, or fibrosis [108,109]. However, Aller et al. reported that circulating visfatin levels may predict portal inflammation, but not steatosis or fibrosis, in non-diabetic obese NAFLD patients [116]. In addition, Kukla et al. found a positive correlation between hepatic visfatin expression and the fibrosis stage but not hepatic steatosis and inflammation in morbidly obese NAFLD patients [112], while Gaddipati et al. reported a positive correlation between visfatin expression in visceral adipose tissue and steatosis degree in non-diabetic NAFLD patients [114].

Interestingly, visfatin was recently proposed as a potential serum biomarker related to the degree of hepatic steatosis and fibrosis among pediatric obese patients diagnosed by non-invasive methods (abdominal ultrasound and transient elastography with liver stiffness and controlled attenuation parameter) [117]. Moreover, a 10-year follow-up study verified no association between serum visfatin levels and leukocyte infiltration in fatty liver at the baseline, but visfatin serum levels were significantly increased during the follow-up, likely due to the combined effects of augmented BMI and diabetes prevalence [118]. However, this study had certain limitations, in particular, the use of ultrasonography as the NAFLD diagnosis method, without confirmation by hepatic biopsy, and the measurement of visfatin levels in all samples (basal and 10-years after) at the end of the study, which likely affects the visfatin concentrations due to different storage times. 

Thus, the different methodological strategies used to study visfatin levels in human NAFLD likely determine the inconsistencies among the current data, and future research is still needed.

### 5.5. Chemerin

Since chemerin regulates insulin signaling and chronic inflammation, it is reasonable to hypothesize that this adipokine may be related to NAFLD development. In fact, elevated serum chemerin levels were identified as a risk factor for NAFLD development in T2DM patients [119] and were pointed out as a novel non-invasive serum marker predicting liver steatosis in obese children [120]. A recent meta-analysis [121] further explored the correlations between serum chemerin levels and NAFLD (steatosis and/or NASH) and its specific hepatic histologic lesions (liver steatosis, lobular and portal inflammation, and fibrosis). Overall, circulating chemerin levels were consistently higher in patients with NAFLD and steatosis compared to controls, although no significant difference was verified between NASH patients and controls. Moreover, data on serum chemerin levels and specific liver lesions are inconsistent, and no correlations were verified [81,121].

It was found that chemerin expression in visceral adipose tissue was negatively correlated with the steatosis score and NAFLD ACTIVITY SCORES of obese NAFLD patients, likely through the modulation of IR and, thus, NAFLD [122]. Data on hepatic chemerin mRNA expression are contradictory; its levels were found to be negatively associated with inflammation, fibrosis, and NAS, but not with steatosis, in non-obese NAFLD patients [123], while other studies verified an increased hepatic chemerin mRNA expression, as well as hepatic CMKLR1 expression, that correlated with hepatic steatosis, hepatocyte ballooning, and the NAFLD activity score in obese NAFLD patients [124,125]. Of note, the hepatic expression of both chemerin and CMKLR1 was associated with obesity [125], which can partially explain the inconsistency of the results.

### 5.6. Adipocyte Fatty Acid-Binding Protein (AFABP)

In ultrasound-diagnosed NAFLD patients, serum AFABP levels were higher in NAFLD patients compared with non-NAFLD group [126,127]. The same was observed in biopsy-proven NAFLD patients, where serum AFABP had an independent positive correlation with lobular inflammation and hepatocyte ballooning, even after adjusting for confounding factors [128,129]. Milner et al. also reported higher serum AFABP levels in NASH patients compared with SS and correlated AFABP with IR, adiposity, and the fibrosis stage [128]. Nevertheless, other studies did not verify an association between AFABP and fibrosis, or that this adipokine was able to distinguish NASH from non-NASH patients [110]. In summary, serum AFABP levels are elevated in NAFLD, but its correlation with NASH, and particular fibrosis, is still unclear.

**Figure 2:**
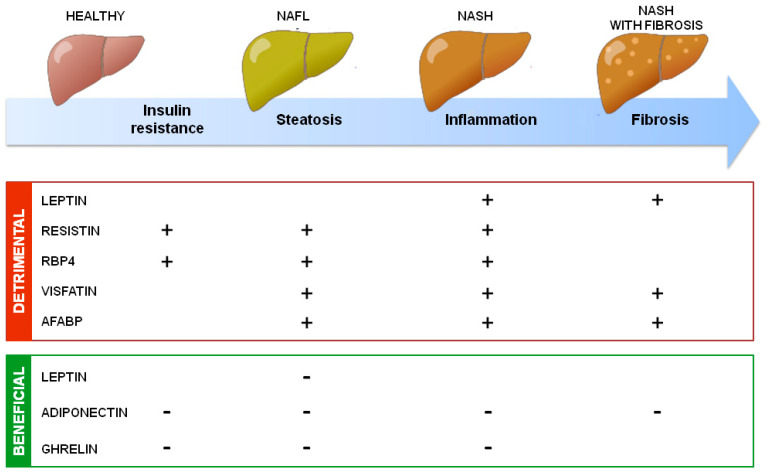
**Summary of adipokines’ action in NAFLD development**. Adipokines secreted by adipose tissue could have detrimental or beneficial effects on NAFLD development through, respectively, increasing (+) or decreasing (−) insulin resistance, hepatic steatosis, inflammation, and fibrosis.

In addition to uncertainties, the general accepted roles of adipokines on NAFLD development are summarized in Figure 2.

## 6. Therapeutic Interventions and Modulation of Adipokines’ Levels

As described above, clinical studies bring out much uncertainty regarding the correlation between adipokine levels and NAFLD. However, small interventional studies have revealed that lifestyle modifications and pharmacologic agents could affect the circulating levels of adipokines in NAFLD patients. Although only a few of these studies have adipokines as their main endpoint and most of them are characterized by a reduced sample size and the absence of control groups, they highlight the importance of adipokines in NAFLD amelioration and so are also summarized here.

Weight loss following bariatric surgery or lifestyle modification, namely healthy eating habits and physical exercise, in the NAFLD population, diminishes circulating leptin [8,22,130,131,132,133], resistin [131], RBP4 [8], visfatin [131], and chemerin [8], while elevating circulating adiponectin [8,131,133,134]. Nevertheless, the direct correlations between hepatic steatosis, weight loss, and adipokine levels have barely been investigated. Recently, the DIRECT PLUS randomized clinical trial verified that a green Mediterranean diet, enriched in specific green polyphenols (Mankai, green tea, and walnuts) and restricted in red and processed meat, led to weight loss, a decline of NAFLD prevalence, and a significant reduction in intrahepatic fat assessed by magnetic resonance, which was associated with a decline in leptin and chemerin plasma levels [135]. Furthermore, a randomized single-center study investigating the effects of guided lifestyle changes with endurance activity and nutrition advising or meal replacement with soy protein-based preparation in NASH patients demonstrated the positive correlation of leptin changes with body weight, fat mass, and reduction in waist circumference and total abdominal fat, while adiponectin changes were inversely correlated with these parameters [136]. More importantly, adiponectin increased with a reduction of intrahepatic lipid content, assessed by magnetic resonance imaging, while leptin declined as the intrahepatic lipid content decreased [136].

Thiazolidinediones (TZDs) are a family of drugs used in the treatment of T2DM [137]. As activators of the peroxisome proliferator-activated receptor γ (PPAR-γ), these drugs improve insulin sensitivity, and, therefore, some clinical trials have evaluated the effect of approved TZDs (pioglitazone and rosiglitazone) on NASH [138,139,140]. Both pioglitazone (30 mg/day for 22 months) [140] and rosiglitazone (8 mg/day for 3 years) [139] clearly reduced hepatic steatosis, whereas pioglitazone further reduced lobular inflammation in non-diabetic NAFLD patients [140], as well as fibrosis in biopsy-proven NAFLD patients with T2DM [141]. Rosiglitazone had no beneficial effects on hepatic lobular inflammation or fibrosis [139]. Additionally, a systematic review found an inverse association between the increase of adiponectin levels and histological steatosis, IR, and liver function markers (ALT and AST) after TZDs treatment [142]. So, TZDs increase adiponectin levels in addition to any weight gain, commonly seen with these drugs and usually associated with reduced adiponectin levels and adverse effects in NAFLD, likely through the induction of adiponectin production and secretion, which counterbalances the increase of body weight induced by TZDs [142]. A recent randomized controlled trial also correlated the amelioration of hepatic steatosis and necroinflammation in NASH patients with the enhancement of adiponectin levels and the decrease of visceral-to-subcutaneous fat ratio after pioglitazone (45 mg/d for 6-months) treatment [143]. Altogether, these results reinforce the role of adiponectin on NAFLD. Regarding the leptin levels, it has been demonstrated that TZDs reduced the leptin expression in adipose tissue, which could counteract the enhancement of leptin levels due to the TDZs-induced adipose tissue increase, with a neutral effect of TZDs on circulating leptin levels in NASH patients [8]. Of relevance, preliminary data revealed that the beneficial effects of pioglitazone on adiponectin levels and hepatic histology in NAFLD patients were reversed after therapy discontinuation, suggesting that long-term therapy with TZDs may be required [144]. However, treatment with rosiglitazone has been restricted due to the increased risk of myocardial infarction [145], whereas the administration of pioglitazone has been halted in some European countries due to its association with bladder-cancer risk after long-term use in diabetic patients [146]. Therefore, TZDs have demonstrated promising results in NAFLD amelioration, mainly through the reduction of hepatic steatosis correlated with increased adiponectin levels, but their clinical use could be limited by poor activity on hepatic fibrosis and long-term deleterious effects. The newer selective PPAR-γ modulators (SPPARMs) have been identified as promising agents that could provide better outcomes in NAFLD patients, but long term placebo-controlled randomized trials are still needed [147].

Another drug extensively used as a first-line therapy in T2DM is metformin, because of its outstanding glucose-lowering properties and its ability to improve insulin sensitivity [148]. So, it is not surprising that the therapeutic activity of metformin in NAFLD has been investigated. Preclinical data found that metformin prevented NAFLD development through the suppression of hepatic TG accumulation [149]. However, the data of clinical trials on metformin activity in NAFLD patients are not so promising. Biochemical parameters, including fasting plasma glucose, postprandial glucose, HOMA-IR, hemoglobin A1c, AST, ALT, GGT, TG, and total cholesterol, as well as body weight, BMI, and waist-to-hip ratio, all significantly improved with metformin therapy comparing to the control group; however, liver histologic scores, namely steatosis, ballooning, NAFLD activity score, and fibrosis, did not significantly change after metformin treatment, and lobular inflammation even became worse after therapy [150,151,152,153]. The beneficial effects of metformin on biochemical parameters and body weight in NAFLD patients were parallel to an increase in adiponectin levels [152,153] and a reduction in chemerin levels [151]. Interestingly, the one-year treatment with metformin decreased the central aortic augmentation index, which increased circulating adiponectin levels, an independent predictor of arterial stiffness improvement, which indicates that metformin therapy could ameliorate vascular dysfunction in NAFLD patients [154].

In the last few years, sodium-glucose cotransporter-2 (SGLT2) inhibitors, namely, empagliflozin, dapagliflozin, ipragliflozin, luseogliflozin, canagliflozin, and tofogliflozin, have demonstrated beneficial effects in T2DM patients with NAFLD. These blood–glucose-lowering drugs can significantly reduce hepatic enzymes and hepatic fat and improve metabolic and fibrosis indexes without increasing adverse effects [155,156]. The effect of SGLT2 on adipokine levels was poorly explored in clinical trials with NAFLD patients. A study verified no significant differences in adiponectin and leptin levels in the dapagliflozin-treated group compared to the placebo group [157], whereas the lower serum level of high-molecular-weight adiponectin was correlated with an improved response to dapagliflozin in T2DM patients with NAFLD [156]. Altogether, SGLT2 inhibitors seem to be an interesting therapeutic option for T2DM patients with NAFLD, but clinicians need to consider their efficacy and safety when individualizing the treatment [156].

Despite the modulation of adipokine levels by therapeutic approaches used in the management of NAFLD and its comorbidities T2DM and obesity, the effect of direct adipokine targeting in NAFLD has been poorly investigated. As described above, increased circulating levels of adiponectin seem to have beneficial effects in NAFLD patients, and, so, its administration could be seen as an appealing strategy for NAFLD. However, adiponectin is subjected to extensive post-translational modifications and multimerization, which challenges the production of functionally active recombinant adiponectin [158]. This is why the discovery of adiponectin analogues, such as osmotin, a plant antifungal protein, might result in alternative therapies for NAFLD [159,160]. Another alternative therapeutic approach for NAFLD in patients with remaining functional adipose tissue could be the up-regulation of endogenous adiponectin expression and/or its secretion induced by sustained weight loss or pharmacological agents, such as TZDs or SPPARMs, as described above [5]. The only “adipokine drug” approved by the United States Food and Drug Administration (FDA) is recombinant human leptin—metreleptin, used to treat complications of leptin deficiency in patients with lipodystrophy [161]. Data from small interventional studies verified a reduction in hepatic steatosis and improvements in hepatocellular ballooning and NAS, but not fibrosis, in NASH patients with a relative leptin deficiency or partial lipodystrophy [162,163,164]. So, although large well-controlled studies are needed, it seems that leptin treatment could become a therapeutic tool for hypoleptinemic NAFLD patients. Moreover, the discovery of leptin analogues with anti-steatotic action but lacking the inflammatory and fibrogenic leptin activity, such as 7i [165], will be of relevance in the development of adipokine-based therapies for NAFLD [22].

## 7. Future Perspectives and Conclusions

The Global Burden of Disease Study 2017 evidenced the alarming trend of NAFL/NASH to become the major cause of chronic liver disease in children and adults and to potentially contribute to the burden of non-communicable diseases, such as cardiovascular disease [2]. Therefore, the early screening of NAFLD patients at highest risk for liver-related complications and the development of successful management strategies are mandatory. The emerging roles of adipokines as metabolic and inflammatory players, together with encouraging preclinical data, have identified adipokines as promising biomarkers and therapeutic targets for steatosis and NASH. Nevertheless, the available clinical data are inconsistent.

Every clinical study has a unique set of inclusion and exclusion criteria for participants. A considerable number of clinical trials used magnetic-resonance-imaging-based NAFLD diagnosis without liver biopsy confirmation or histological NAFLD grade evaluation, which led to highly heterogeneous groups and limited the conclusions of these studies. NAFL and NASH classifications are still widely used in clinical practice, but the recent definition of MAFLD will allow a better characterization of patients’ profiles, excluding those with steatosis unrelated to metabolic dysfunction and, thus, contributing to more homogeneous cohorts [4]. Moreover, the establishment of diagnosis and follow-up guidelines [1,11,166] as effective tools to identify and stratify NAFLD patients is helpful. Moreover, the pathogenic factors associated with NAFLD are multifactorial, including lifestyle (high-calorie diet and sedentarism), genetic alterations, or the presence of comorbidities [21], and the analysis of possible confounding factors are critical. In this context, future clinical trials with highly-phenotyped patient cohorts, similarly to the European NAFLD Registry [167], are demanding.

The existing clinical studies on adipokines and NAFLD are mostly case-control or cross-sectional studies, which limited the conclusions regarding causalities and possible associations. Prospective cohort studies with paired biopsies, long-term follow-ups and carefully matched controls can offer a higher level of evidence on adipokines’ pathophysiological role in NAFLD and on their diagnosis and prognosis power. In this regard, it will be important to define standardized methodology for high-sensitivity, rapid, single-analysis, and, most importantly, the reliable identification and quantification of adipokines [168], without forgetting that some of them have multiple forms, such as adiponectin, resistin, and ghrelin [26,42,158], or can be found in the circulation as free or protein-bounded forms, such as leptin [169]. Moreover, the levels of endogenous antagonists of adipokines [170] or inhibitors of their receptors should not be ignored. Finally, increasingly important topics are proper clinical study design, analysis, reporting, and sharing of data, to ensure accurate and robust findings as well as appropriate interpretation [171,172].

As described in this review, the potential role of adipokines in NAFLD development has been broadly explored using in vitro and in vivo approaches. While cellular models lack systemic perspective and cross-organ communication, current NAFL/NASH in vivo models have limited predictive value to the full spectrum of human NAFLD [173]. However, nutritional models or combined models of genetic modifications and nutritional challenges presenting metabolic comorbidities (IR, dyslipidemia and obesity) [173] are likely to be the best-suited models with a higher translatable potential for the study of adipokines’ pathophysiological role in NAFLD. Furthermore, recent advances on 3D cell cultures and biochip-based culture systems [174] could allow a better assessment of cellular responses to adipokines. Altogether, preclinical models are useful tools to unravel pathophysiological links and signaling pathways, which could be very valuable in disclosing uncertainties on the role of adipokines in NAFLD. Curiously, there are limited studies either on the hepatic functionality of adipokines receptors or on the activity of endogenous adipokines antagonists in NAFLD, which could add other (dys)regulatory levels to the intricate communication between adipose tissue and the liver. Furthermore, several emerging adipokines, such as vaspin [175,176,177], adipsin [178], apelin [179,180], obestatin [181], and omentin [176,177], have been associated with NAFLD, but their roles and mechanisms of action remain unclear.

Future insights into the pathophysiologic role of adipokines in NAFLD will be of great relevance for the development of new therapeutic approaches. In particular, the control of bioactive adipokines levels using high-affinity binding molecules, miRNAs targeting specific adipokines, and antagonists or monoclonal humanized antibodies against adipokines receptors are likely to be feasible options [182]. Accordingly, as mentioned above, recombinant leptin was described to improve steatosis and NAFLD activity score in hypoleptinemic NAFLD patients [162,163,164]. However, there are some concerns to take into account when targeting adipokines or their receptors. Anti-drug antibodies, developed after adipokine administration, could cross-react with endogenous adipokine and limit their therapeutic efficacy [183]. Furthermore, given the pleiotropic activity of adipokines, existence of compensatory mechanisms, and interplay among detrimental and beneficial adipokines, a systematic approach might be discouraged; instead, strategies targeting specific receptor isoforms or adipokines activity precisely in specific cell populations could be viable therapeutic options [6,8]. The development of adipokines analogues without deleterious effects, such as the leptin analogue 7i [165] are also an attractive strategy. Above all, adipokines are immunometabolic players [7] acting in a dose-dependent and continuous dynamic cross-talk way, and, thus, therapies aiming to reestablish a healthy adipokine network and adipose tissue function are likely to be more effective than single-adipokine targeting.

In conclusion, much uncertainty still exists regarding the roles of adipokines in NAFLD, mainly due to complex NAFLD pathophysiology and intricate adipokines network. Long-term prospective studies of well-characterized cohorts presenting paired liver biopsies and suitable matched controls, together with improved preclinical models, are demanding to accurately evaluate adipokines as NAFLD biomarkers as well as adipokine-based therapies for NAFLD. Through the path, strict collaboration between clinicians and basic scientists will certainly elicit cross-fertilization of translational research and accelerate the transfer of the results to the clinical practice. Nevertheless, we should keep in mind that the more effective approach to counteract adipokine imbalance and NAFLD is to prevent and/or to revert excessive fat.

## Figures and Tables

**Table 1:** Overview of adipokines.

**Adipokine**	**Description**	**Receptor/Signaling**	**Functions**	**Refs.**
Leptin	cytokine-like hormone encoded by LEP gene (obese gene, ob). secreted by WAT, brain, intestines, skeletal muscle, placenta, etc.	LEP-R (encoded by LEPR), which have at least six isoforms LEP-R long isoform signals via JAK–STAT activation or, alternatively, via p38 MAPK, JNK, ERK1/2, PI3K/Akt or PKC signaling	controls appetite and body weight at the hypothalamus level regulates insulin secretion, thermogenesis, lipid homeostasis, reproductive functions, inflammation, infection, angiogenesis, and homeostasis of cartilage and bone	[6,22,23]
Adiponectin (ACRP30, AdipoQ, GBP28 or apM1)	encoded by ADIPOQ gene homologous to C1q, collagen VIII and collagen X 12–18-monomers, trimers, and hexamers forms produced by AT, bone marrow, skeletal muscle, and cardiac tissue	AdipoR1 (mainly present in skeletal muscle) and AdipoR2 (prevalent in the liver) signals through AMPK, PPARα or PPARγ	augments FA oxidation and glucose uptake in the muscle decreases glucose synthesis in the liver affects obesity, metabolic syndrome, lipodystrophy, and cardiovascular disease.	[24,25]
Ghrelin	hormone expressed by the stomach’s oxyntic glands, pancreatic islets, hypothalamus, lung, testis, and ovary	acylation catalyzed by GOAT (UAG to AG conversion) AG activates, while UAG antagonizes, GHSR1a	triggers food intake and adiposity regulates glucose metabolism, reward behavior, gut motility, and immune system	[26,27]
Resistin (ADSF or FIZZ3)	found as dimers in human blood produced in macrophages, mononuclear leukocytes, bone marrow cells, and spleen	ROR-1, IGF-1R and CAP1 are potential receptors toll-like receptor (TLR) 4 mediate resistin-induced pro- inflammatory cytokines secretion via NF-κB and C/EBPβ.	regulates blood glucose levels and lipid metabolism and promotes IR induces pro-inflammatory cytokines secretion and monocytes differentiation into macrophages.	[28]
RBP4	member of the lipocalin family expressed by liver, AT, retinal pigment epithelium, kidney, peritubular, and Sertoli cells of the testis bound to TTR in the circulation	STRA6 mediates retinol influx from the blood to target cells	transports retinol (essential for the visual cycle) contributes to IR, dyslipidemia, T2DM and cardiovascular dysfunction	[29]
Visfatin (PBEF or NAMPT)	homodimeric cytokine-like peptide with intracellular (iNAMPT) and extracellular (eNAMPT) forms	unidentified specific receptor iNAMPT is the rate-limiting enzyme of NAD biosynthesis from nicotinamide.	iNAMPT modulates cellular metabolism, differentiation, and stress response eNAMPT induces pro-inflammatory cytokines production and associates with metabolic and inflammatory diseases	[30,31]
Chemerin (TIG2 or RARRES2)	secreted as an inactive precursor (prochemerin) activated by proteases of the coagulation cascade, neutrophil-derived proteases (elastase and cathepsin G), bacterial proteases, and mast cell products (tryptase)	CMKLR1 mediates chemerin’s chemotactic activity GPR1 and CCRL2 also binds chemerin, but their functional relevance is unknown	Regulates adipocyte differentiation, insulin sensitivity, glucose, and lipid metabolism bridges innate and adaptive immunity through CMKLR1 (expressed in antigen-presenting cells, natural killer cells, and macrophages)	[32]
AFABP (ap2 or FABP4)	belongs to the lipocalin family abundant cytosolic protein of mature adipocytes also produced by endothelial cells and macrophages	unidentified receptor high affinity and selectivity for long-chain fatty acids induced by FA, TLRs agonists, oxLDL, and advanced glycation end products	modulates lipolysis in adipocytes promotes cholesterol esters accumulation and foam-cell formation induces endothelial dysfunction	[33]
